# Supplementary figures and images for: Chromatin profiling reveals heterogeneity in clinical isolates of the human pathogen Aspergillus fumigatus
Source: PLoS Genet. 2022 Jan 10;18(1):e1010001. doi: 10.1371/journal.pgen.1010001 (PMC8782537; doi:10.1371/journal.pgen.1010001)

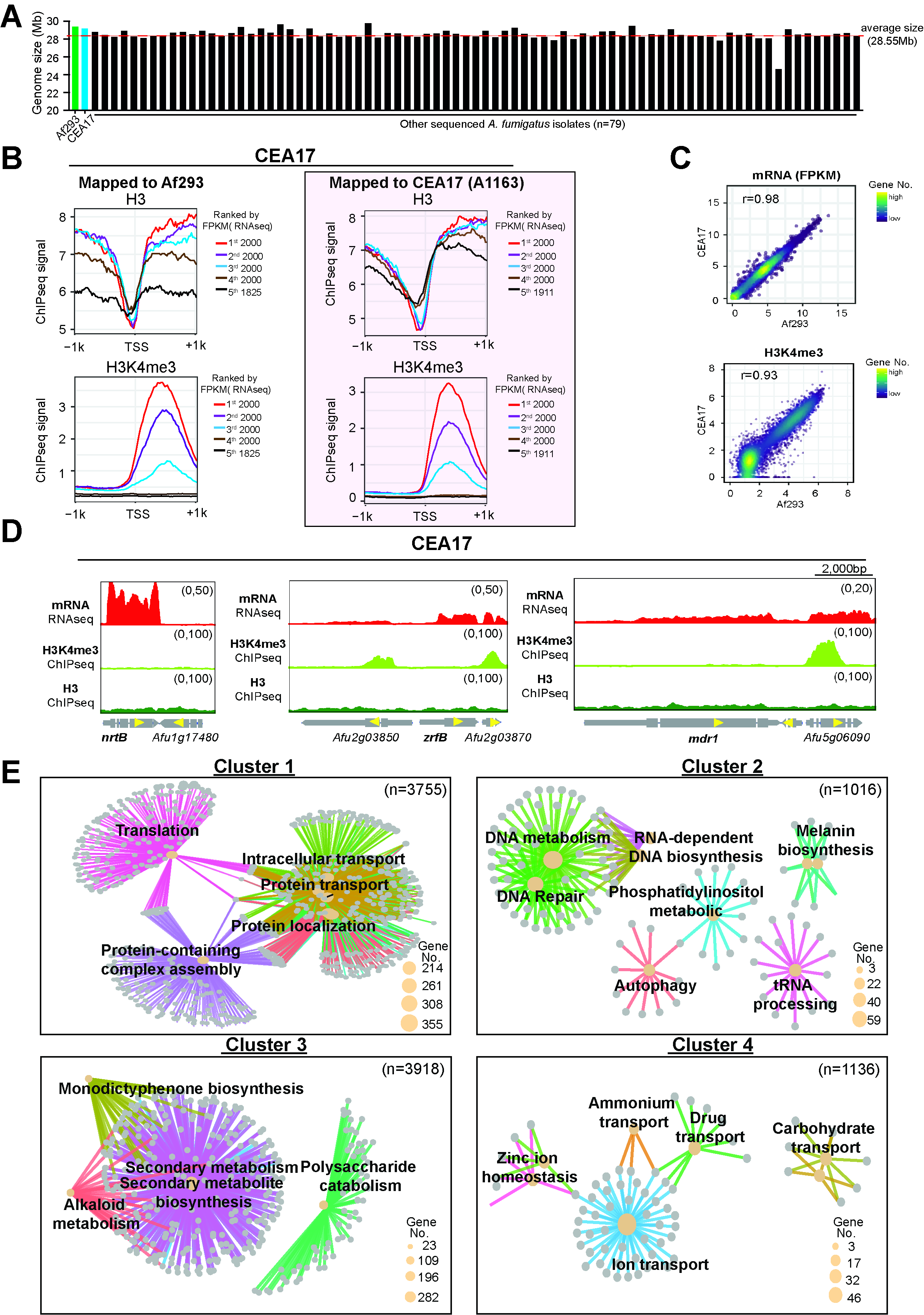

Supplement: S1 Fig — (A) A bar plot showing the genome size of Af293, CEA17 and the sequenced A. fumigatus isolates described in [64,65]. (B) Line plots showing the H3 and H3K4me3 deposition within 1 kb of TSS region of genes genome wide in CEA17 when the data was mapped to Af293 and CEA17 (A1163) reference genome, respectively. Gene order was ranked by mRNA level from high to low. The pink shade was plotted as shown the mapping was performed to CEA17 genome reference. (C) Scatter plots showing the correlation of mRNA level (upper panel) and H3K4me3 deposition level (bottom panel) in Af293 and CEA17. (D) Genome browser screenshots showing the mRNA and H3K4me3 level at selected genes in CEA17. H3 was used as control. (E) Gene Ontology analysis of gene sets in cluster 1–4 as shown in Fig 1E-F. (TIF) [file pgen.1010001.s001.tif]

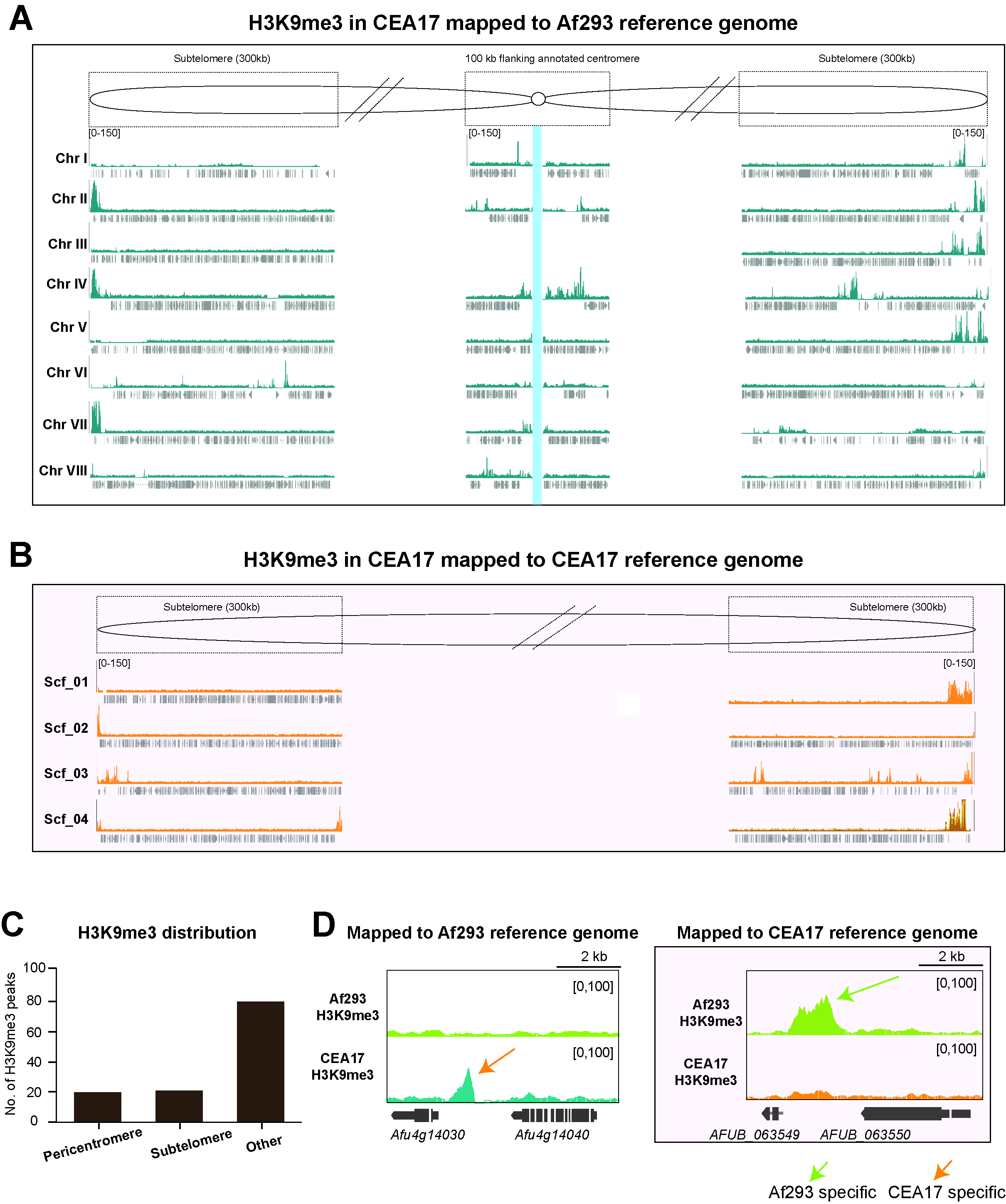

Supplement: S2 Fig — (A) A genome browser screenshot showing the H3K9me3 ChIPseq signals in the subtelomeric and pericentromeric regions in CEA17 isolate when mapped to Af293 genome reference. Blue shade was marked with the annotated centromere region. (B) Genome browser screenshots showing the H3K9me3 ChIPseq signals at the two ends (300 kb) of four CEA17 contigs. (C) A bar plot showing the number of H3K9me3 peaks located in pericentromeric (n = 20), subtelomeric (n = 21) and other genomic regions (n = 80). (D) Genome browser screenshots showing the H3K9me3 profile of regions with dissimilar modification between Af293 and CEA17. Arrows mark the loci with different (green and orange) H3K9me3 depositions in two isolates. The pink shade in (B) and (D) represents the mapping was performed to CEA17 genome reference. (TIF) [file pgen.1010001.s002.tif]

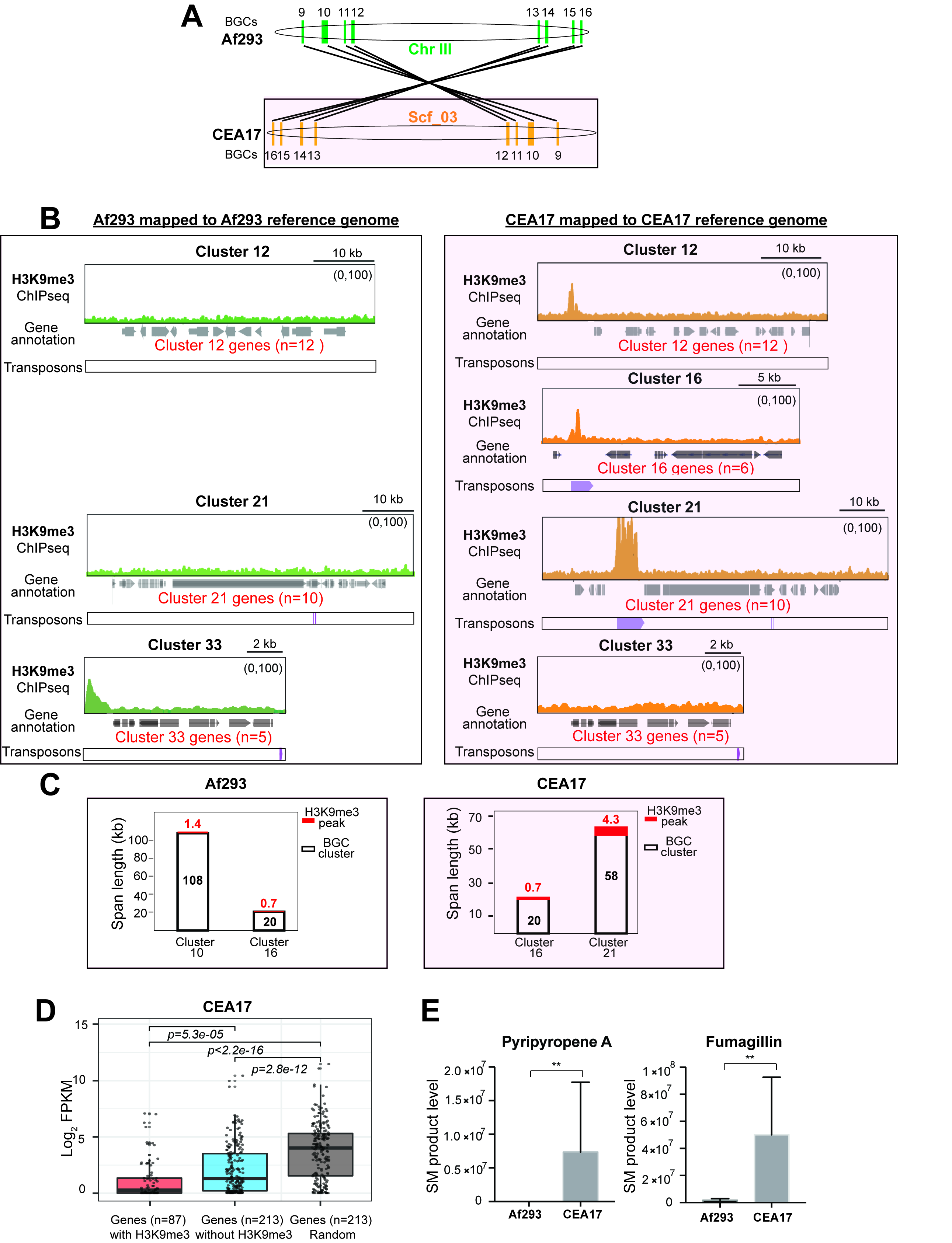

Supplement: S3 Fig — (A) A scheme diagram showing the inverted distribution of BGCS 9–16 in Af293 Chr III and CEA17 Scf-03. (B) Genome browser screenshots showing the selected BGCs [BGC 12, 16, 21 and 33] marked with H3K9me3 in Af293 [BGC 12, 16 and 21] or CEA17 [BGC 33]. TEs were indicated with purple markers in (A) and (B). (C) Bar plots showing the span length of H3K9me3 peak along with its bound BGCs in Af293 and CEA17. (D) A bar plot showing the expression of BGC genes with (red) or without (blue) H3K9me3 in CEA17. Random remaining non-BGC genes (grey, n = 213) were plotted as control. (E) Bar plots showing the quantified production of SMs pyripyropene A and fumagillin in Af293 and CEA17. The SMs levels were calculated as peak area, and P values were calculated by F test to compare variations. Error bar represent standard derivation and * means P value <0.05; ** means P value <0.01; *** means P value <0.001, **** means P value <0.0001. The pink shade in (A), (B) and (C) is to distinguish data mapping to the CEA17 genome reference. (TIF) [file pgen.1010001.s003.tif]

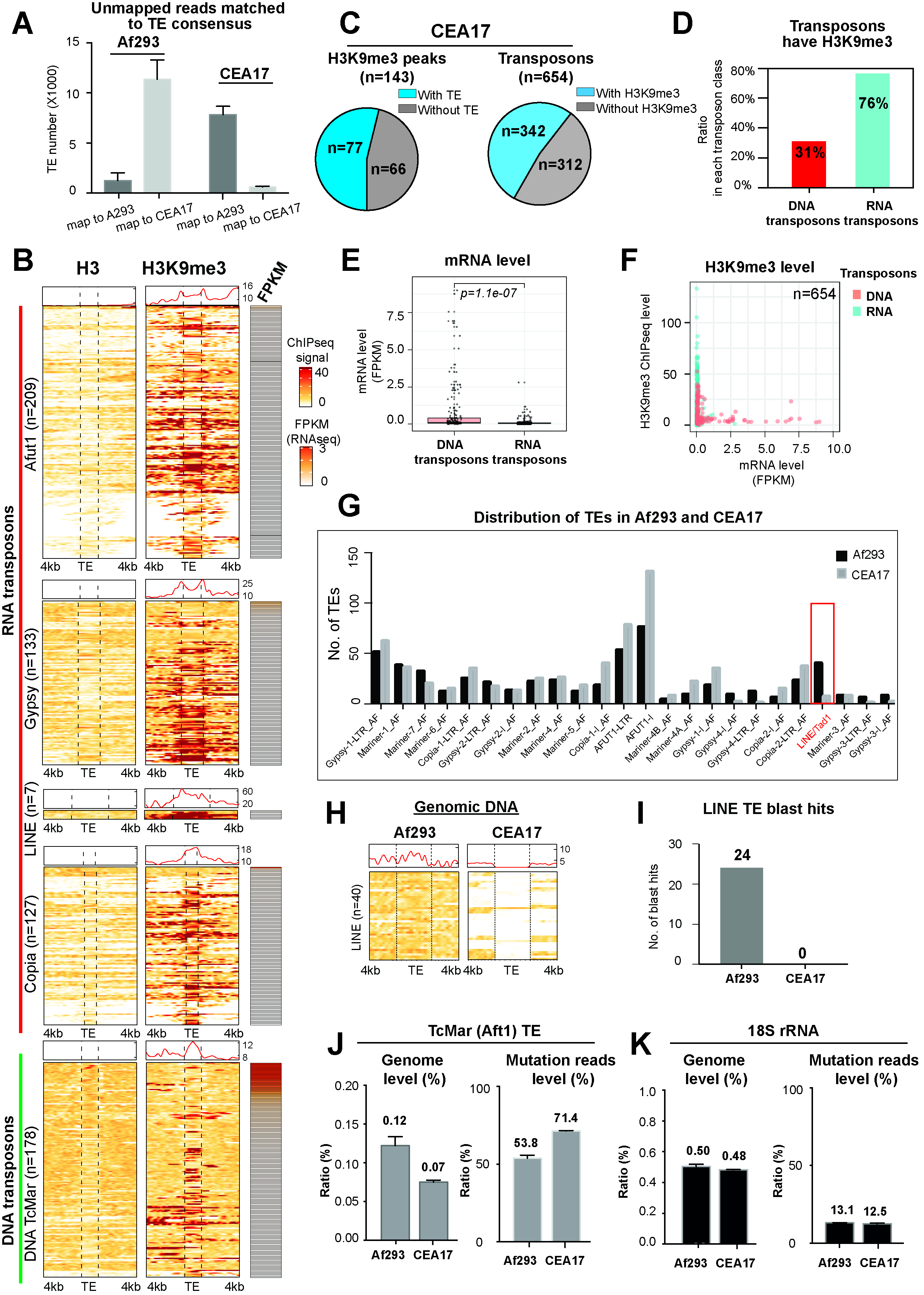

Supplement: S4 Fig — (A) A bar plot showing the number of unmapped reads (from Bowtie alignment to the indicated reference genome) matched to TE consensus by Repeatmasker. Error bar represent standard derivation of two biological replicates. (B) Heatmap plots showing the H3 and H3K9me3 deposition at CEA17 transposon loci identified by Repeatmasker and at their boundary regions (+/- 4 kb) and their expression values (in FPKM). (C) Sector diagrams showing the distribution of H3K9me3 peaks with or without TEs (left panel), and transposons with or without H3K9me3 (right panel) genome wide in CEA17. (D) A bar plot showing the number of DNA and RNA transposons bound by H3K9me3 in CEA17. (E) A bar plot showing the expression level of DNA and RNA transposons in CEA17. (F) A scatter plot showing the relationship between expression level and H3K9me3 modification level at DNA and RNA transposons in CEA17. (G) A bar plot showing the distribution in families of Af293 and CEA17 TEs according to Repeatmasker classification. (H) Heatmap plots showing the LINE transposon level in Af293 and CEA17. (I) A bar plot showing the number of blast hits for LINE transposon in Af293 and CEA17. (J-K) Bar plot showing the genome percentage and mutation reads percentage of (J) Aft1 transposons and (K) 18S rRNAs in Af293 and CEA17. Error bar represent standard derivation of two biological replicates. (TIF) [file pgen.1010001.s004.tif]

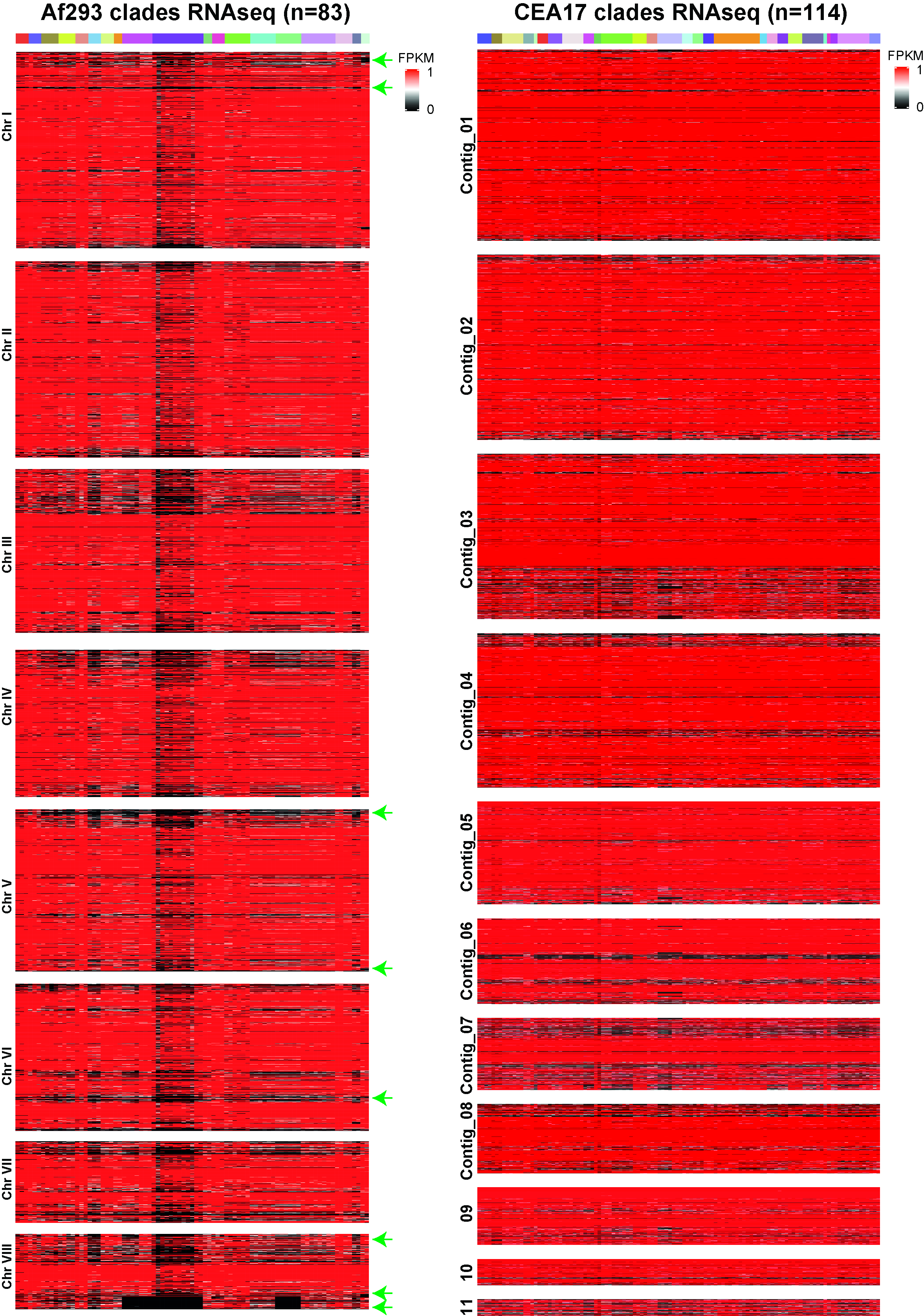

Supplement: S5 Fig — Heatmaps showing the mapped reads in genes of all chromosomes of Af293 clade data (n = 83) and 11 contigs of CEA17 clade data sets (n = 114) from 19 and 28 studies, respectively. Green arrows indicate the genome regions displayed in Fig 6A. (TIF) [file pgen.1010001.s005.tif]

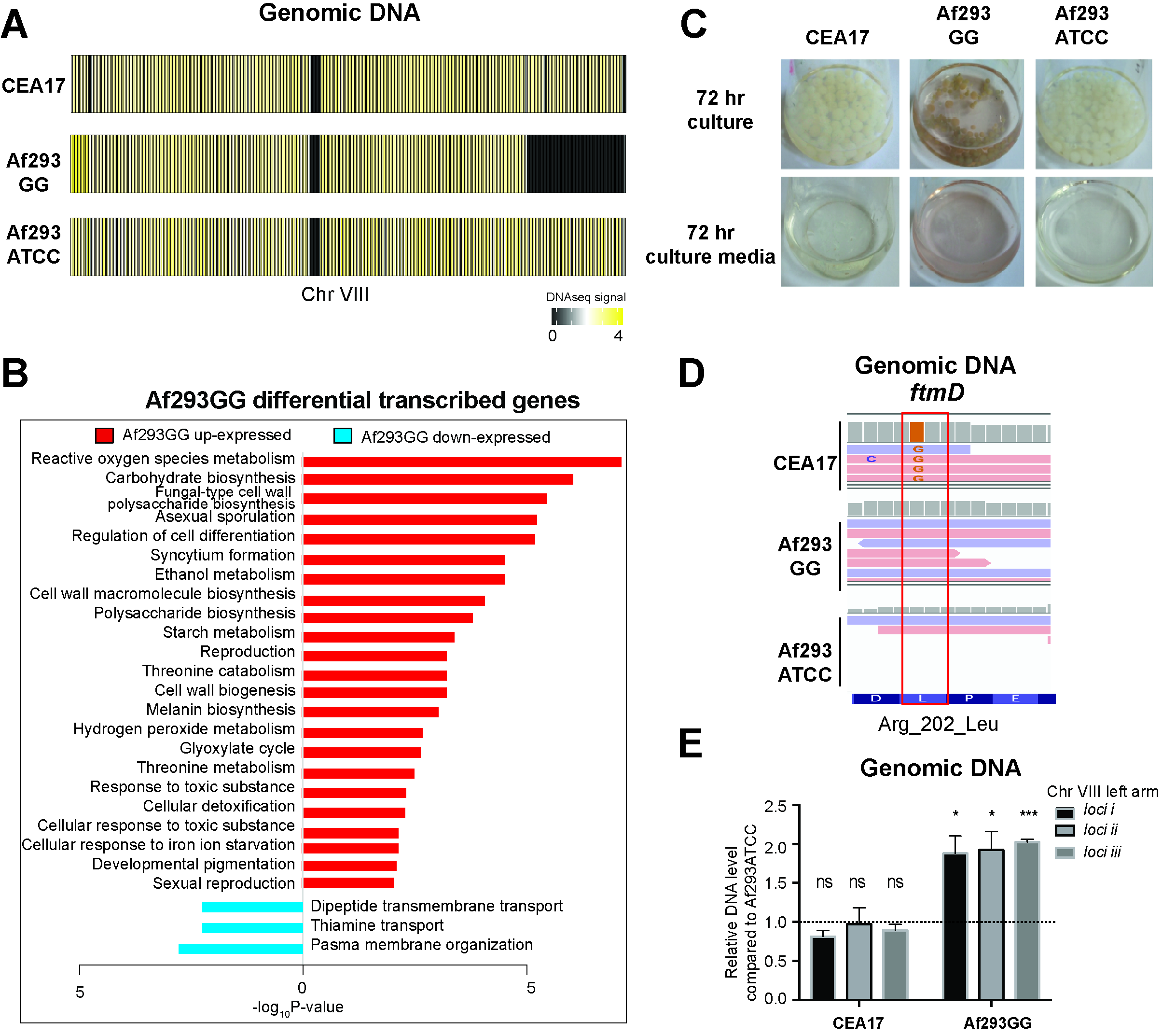

Supplement: S6 Fig — (A) A heatmap plot showing RNA-seq mapped read density to Chr VIII of Af293 genome reference for the CEA17, Af293GG and Af293ATCC strains. (B) A scheme diagram plot showing the Gene Ontology result of Af293GG up and down-expressed genes measured by RNAP II ChIPseq. (C) Photos showing the 72 hours culture and culture media of CEA17, Af293GG and Af293ATCC. (D) A genome browser screenshot showing the SNP mutation of R202L in Af293GG and Af293ATCC isolates compared to CEA17. (E) A bar plot showing qPCR analysis on the selected genomic loci at the Chr VIII left arm in CEA17, Af293GG and Af293ATCC. Data was plotted as normalized values to Af293ATCC. The qPCRs were processed in two independent replicates and P values were calculated by unpaired t test. Error bar represent standard derivation and * means P value <0.05; ** means P value <0.01; *** means P value <0.005. (TIF) [file pgen.1010001.s006.tif]

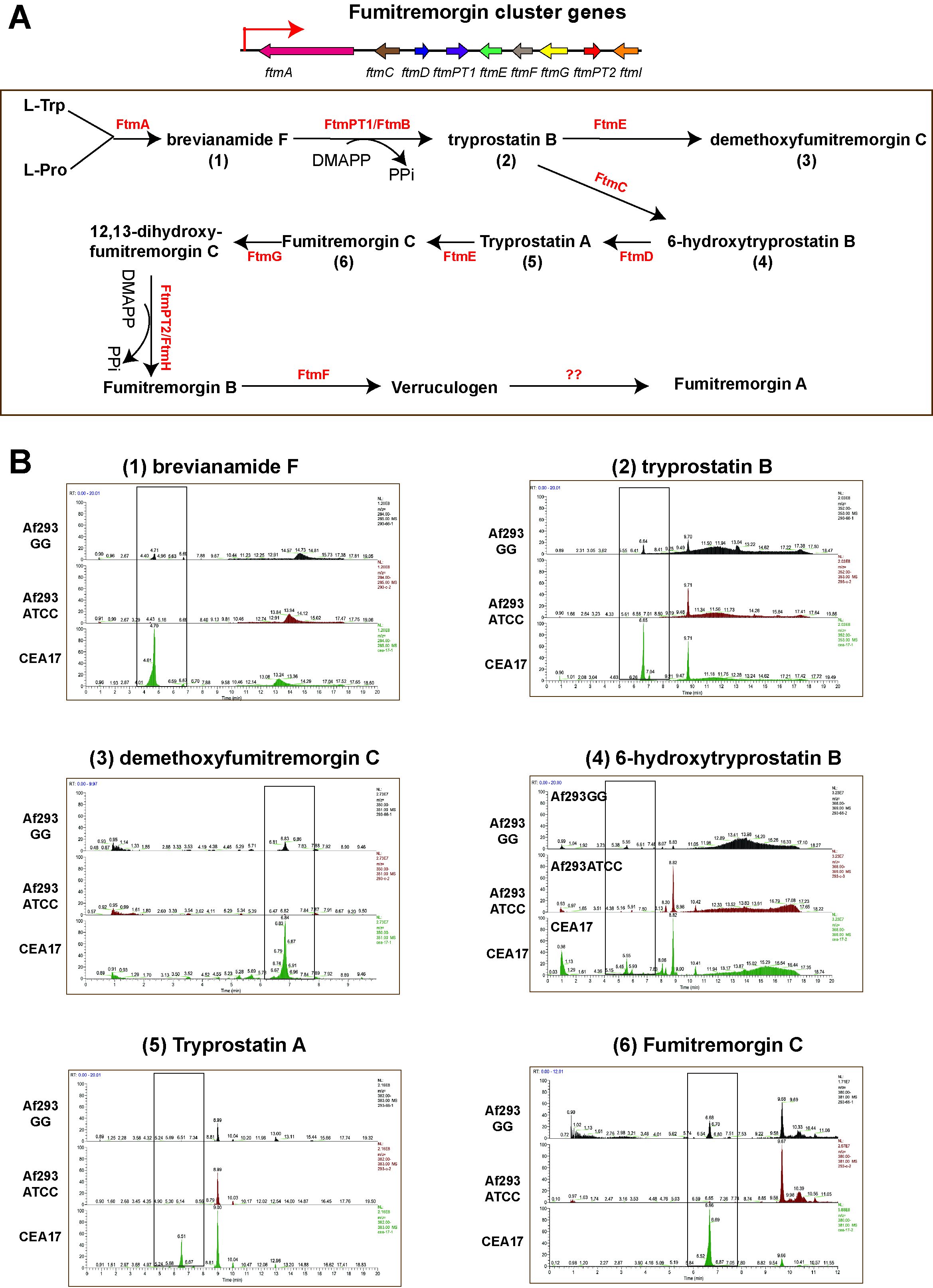

Supplement: S7 Fig — (A) A scheme diagram showing the (top panel) genome distribution of fumitremorgin cluster (BGC 29) genes and (bottom panel) pathway of fumitremorgin A-C biosynthesis. (B) The LC/MS profile of compounds 1–6 in the supernatants of in Af293GG, Af293 and CEA17 strains as shown in (A) and Fig 7J. (TIF) [file pgen.1010001.s007.tif]

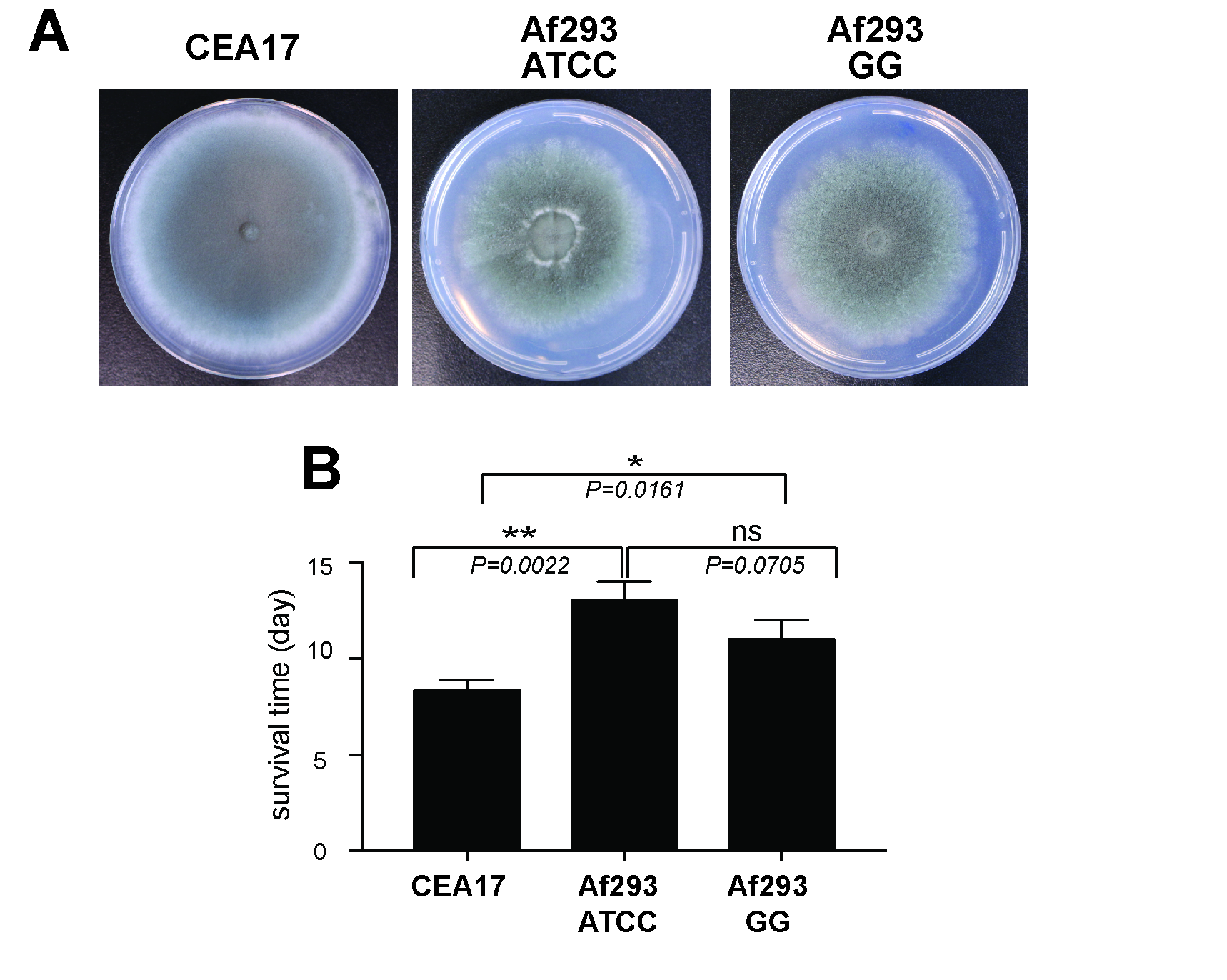

Supplement: S8 Fig — (A) Photos showing the different colony morphologies of CEA17, Af293ATCC and Af293GG isolates. (B) A histogram plot showing the median survival days of the larvae as shown in Fig 7M. Error bar represent standard derivation of three replicates and * means P value <0.05; ** means P value <0.01. (TIF) [file pgen.1010001.s008.tif]

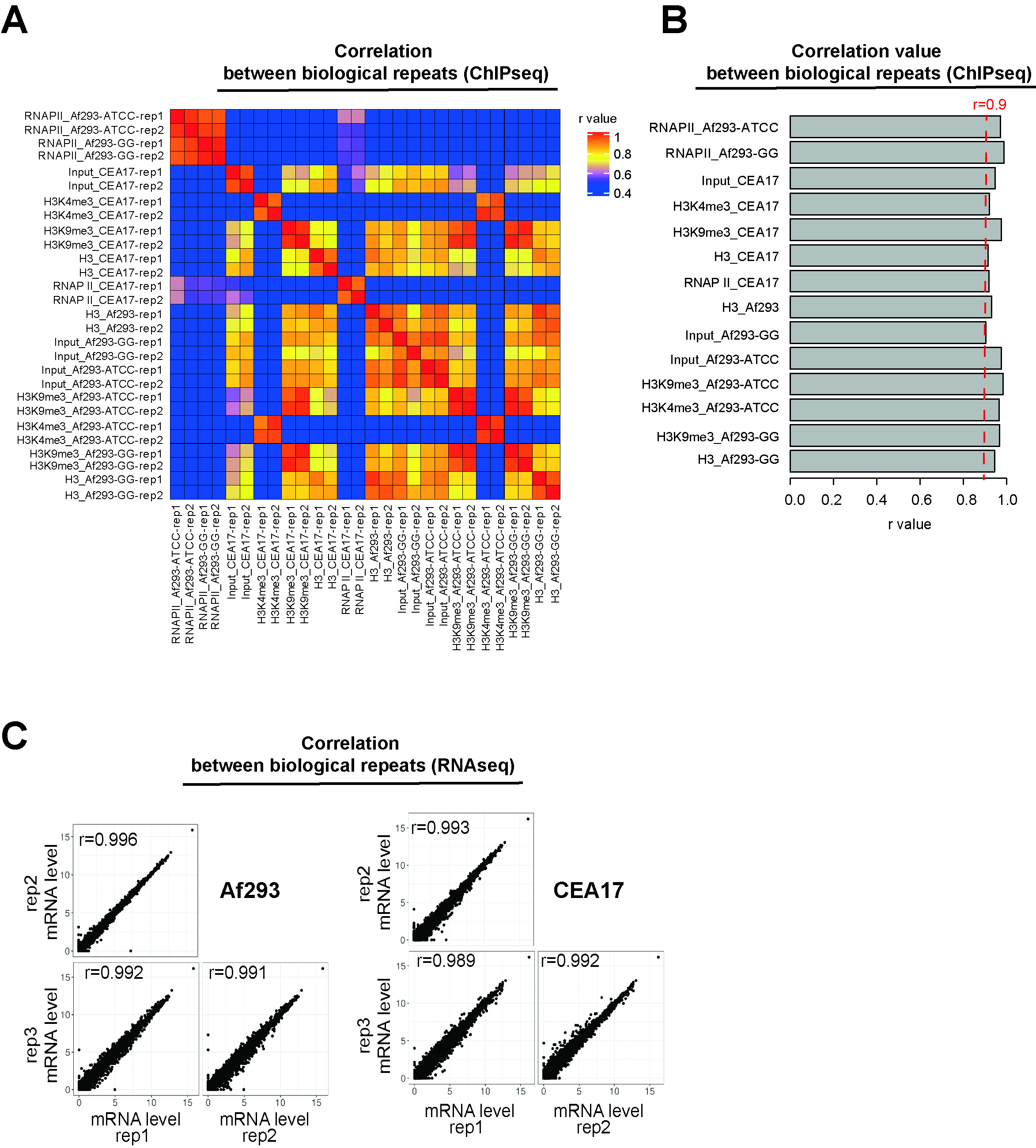

Supplement: S9 Fig — (A-B) A heatmap (A) and bar plot (B) showing the correlation of ChIP-seq biological replicates. (C) A scatter plot showing the correlation of RNA-seq biological replicates. (TIF) [file pgen.1010001.s009.tif]
