# Supplementary material for: Chromatin profiling reveals heterogeneity in clinical isolates of the human pathogen Aspergillus fumigatus
Source: PLoS Genet. 2022 Jan 10;18(1):e1010001. doi: 10.1371/journal.pgen.1010001 (PMC8782537; doi:10.1371/journal.pgen.1010001)
Supplement: Attachment — Submitted filename: Response to the Reviewers_final.docx [file pgen.1010001.s027.docx]

**Response to Reviewers’ comments**

Reviewer #1:

The manuscript ‘Chromatin profiling reveals genome stability heterogeneity in clinical isolates of the human pathogen Aspergillus fumigatus’ reports on chromatin profiles for activating (H3K4m3) and repressing (K3K9me3) histone modifications in two A. fumigates isolates. The manuscript shows differences in transposable elements (TEs) and in genome instabilities, the latter contribute to fitness and virulence.

Overall, the quality of the manuscript is good, and it reports on an exciting suite of novel data that will be interesting for researchers working on Aspergillus, chromatin biology, and genome evolution, among others. However, I have few major concerns related to the experimental approaches and results, and to which extent the data robustly supports the manuscript’s main conclusions.

**Response:** We thank the reviewer for the positive comments and the suggestions to improve the manuscript.

Major remarks:

1) The manuscript shows a) differences in H3K9me3 and TE abundance between different strains, b) differential abundance and activity of specific TEs (LINEs), and c) that strains harbor genomic instability. Based on these observations, the authors conclude that ‘Overall, our work emphasizes the importance of epigenetic modifications in A. fumigatus strain heterogeneity as evolutionary drivers of altered gene expression and genome stability’. While these observations are very interesting, e.g., the chromosomal duplication of chr.8, and clearly correlated, the causative link between chromatin and genome instability has not been established. Knock-out experiments (coupled with ChIP-/RNA-seq) aimed to remove H3K9 methylation are necessary to demonstrate that TE activity is suppressed by H3K9m3 and can be re-established in the absence of this chromatin modification. Furthermore, RNA-seq and H3K9m3 ChIP-seq under different conditions would firmly establish whether TEs, as previously demonstrated for instance for Z. tritici (Fouche et al. 2020), and especially LINEs are displaying differential and H3K9me3 dependent activity (this would also be relevant for the expression of telomere-localized genes, see below). Lastly, the link between H3K9me3 to genome stability needs to be experimentally established. Similar to recent work in in Z. tritici (Möller et al. 2019), Aspergilli with altered H3K9me3 needs to be grown for an extended period of time and re-sequenced to show that i) TEs are re-activated (see above) and ii) that genome instability is increased compared with wild-type strains. In line with this concern, the manuscript’s title does not reflect the conclusions that can be safely drawn from the presented data and analyses, and thus should be toned done at least.

**Response:** We thank the reviewer for these comments. The aim of this paper is to identify and report epigenetic and genetic heterogeneities between the commonly used *A. fumigatus* isolates. The reviewer is right that the suggested experiments are needed to convincingly demonstrate a causative link between chromatin and genome instability. However, we think that the suggested experiments are beyond the aims of our current manuscript. In fact, the function of H3K9me3 is being investigated and will be the subject of a subsequent manuscript. Nevertheless, the reviewer is right that we need to tone down some of our main conclusions, and these are the major changes regarding this (which are the same as the response to Reviewer #2 comment 8):

1) The title is changed from “Chromatin profiling reveals genome stability heterogeneity in clinical isolates of the human pathogen *Aspergillus fumigatus*” to “Chromatin profiling reveals heterogeneity in clinical isolates of the human pathogen *Aspergillus fumigatus*” to emphasize on the findings of epigenetic and genetic heterogeneities between isolates.

2) The conclusion “Overall, our work emphasizes the importance of epigenetic modifications in *A. fumigatus* strain heterogeneity as evolutionary drivers of altered gene expression and genome stability” is deleted from the manuscript.

3) The manuscript has been significantly rewritten to tone down the conclusions according to this reviewer’s comment including (but not limited to) the following examples to just name a few changes:

- Removed sections like “Suggesting that some of them may not be subjected to H3K9me3 silencing since the LINE-transposons are the most active RNA transposons and have a major impact on the genome stability (Bourque *et al*., 2018), These results imply that Af293 genome may have higher LINE-transposon activity and, hence, more unstable”;
- Renamed the title of the sub-section from “Evidence for Af293 having a higher level of genome instability” to “Evidence for Af293 having an increased number of chromosomal rearrangement events”;
- Replaced the sentence “…the induced LINE-transposon activity may be responsible for the chromosomal loss” with “…the induced LINE-transposon activity may be linked to the chromosomal loss”;
- Replaced the sentence “…evidence that the Af293 genome is relatively unstable compared to CEA17” with “…evidence that the Af293 chromosomes are more prone to rearrangements compared to CEA17”;
- Added the following two paragraphs to the Discussion section to clarify the suggestive nature of our findings and highlight the need for additional experiments to confirm the causative links: “*Another interesting finding is that Af293 contains significantly more LINE-transposons than the CEA17 isolate, which was also reported in a previous study (Huber and Bignell, 2014). Our analysis revealed that a high percentage (~70%) of LINE-transposons in Af293 were not subjected to H3K9me3 modification* *(Figure 5e) and that the LINE transposon sequences in Af293 contain relatively much less mutations than in those in CEA17 (Figure 5b, c). Based on these, it is tempting to speculate higher transposon activities in the Af293 isolate. In support of the speculation and consistent with the association of transposons activity with chromosome rearrangement and genome instability in different organisms (Zeller et al., 2016; Miousse et al. 2015; Moller et al., 2019; Kondo et al., 2008; Peters et al., 2001; Peng et al, 2009), we indeed detected gross chromosome alteration events including a chromosomal loss hotspot in Chr VIII in Af293 strains of different laboratories but not from different CEA17 strains. This circumstantial evidence leads us to a proposal that the higher number of LINE transposons in Af293 may be linked to the increased incidents of chromosomal rearrangements observed among Af293 strains. It will be interesting to experimentally confirm this (e.g., by microevolution experiments in the absence of H3K9me3 modification and/or with increased transposon activity) and to compare transposon numbers and activities between different clinical isolates, considering chromosomal rearrangement has been shown as a virulence mechanism for the adaptation of pathogenic Candida species to different host niche environments (Poláková et al. 2009, Selmecki et al., 2006).*” and

“*The identification of the Af293GG and other Af293 variants used by different laboratories implies additional levels of complexities in the heterogeneities of A. fumigatus, emphasizing the fascinating ability of this important fungal pathogen to evolve new growth and fitness characteristics that could influence virulence. Our study suggests a potential link between epigenetic heterogeneity (e.g. H3K9me3 modification), TEs, genome instability and genetic heterogeneity. Confirming a causative relationship between these events and identifying additional mechanisms that drive the heterogeneities will guarantee insights into A. fumigatus virulence.*”

2) The manuscript reports on differences in TE numbers between isolates, and many of these TEs occur at sub-telomeric regions (Fig.2/4). Fedorova et al. 2008 have previously reported on genomic differences at (sub) telomeric regions of A. fumigatus by aligning the CEA17 genome to the Af293 reference. It is thus unclear if the observed differences in TE abundances are due to technical (assembly) or biological factors. Therefore, the authors need to address the differences in genome assembly quality before drawing firm conclusions on differences in TE content. Fig. 4h shows the number of TEs in different genomic regions. It would be clearer if the figure would display TEs in relation to the size of the respective region as sub-telomeric regions are much smaller compared with the overall chromosome and consequently enrichment of TEs in sub-telomeric regions is not clearly visible.

**Response:** We thank this reviewer for this critical, reasonable comment, which is also raised by Reviewer #2. A few control analyses have been added to address the differences in genome assembly quality. Specifically, we compared the size of reference genomes of Af293, CEA10 and 79 other *A. fumigatus* isolates. Compared to the fully assembled Af293, the CEA10 reference genome is estimated to be at least 99% complete. It is also the most completed genomes among the analyzed genomes. The comparison is described in L194-199 as “*Comparison of the size of CEA10 reference genome (A1163) with those of Af293 and other A. fumigatus isolates (n = 79 from Takahashi-Nakaguchi et al., 2015; Zhao et al., 2021) shows the CEA10 reference genome size is greater than most isolates (Supplementary Figure 1a), and comparison to the full assembly of Af293 genome suggests that* *the CEA10 reference probably covers more than 99% of A. fumigatus genome (Supplementary Table 1), and therefore, has sufficient coverage for analysis and comparison.*”

In addition, to rule out the possibility that the observation is due to a technical (assembly) factor, we have also performed Whole-Genome Re-sequencing by Illumina for Af293 and CEA17, mapped the data to their respective genomes and counted TE elements from the unmapped reads. The rationale behind this analysis is that if there were many TEs missing from the partial CEA10 reference genome, many unmapped reads from the mapping of Whole-Genome Re-sequencing data (which covers the entire genome) would be expected to carry TEs and the number would be much higher compared to the control experiment using Af293 Whole-Genome Re-sequencing data. The result of this analysis is described in L373-382 as “*Since the CEA10 reference genome is not fully assembled, we first confirmed that the existing sequence and assembly is suitable for TE analysis. To this end, Illumina sequencing for the Af293 and CEA17 isolates was performed, and the raw sequences were mapped to both reference genomes separately. If there were many TEs missing from* *the partial CEA10 assembly, a high number of unmapped reads (from mapping CEA17 data to the CEA10 genome reference) is expected to contain TE sequences. When using the complete Af293 reference genome and Af293 data as a control, the comparison showed that the numbers of TEs from mapping the Af293 and CEA17 Illumina sequences to their respective genomes are similar (Supplementary Figure 4a), indicating that the CEA10 reference genome is just as good as the Af293 one for TE analysis.*”

Based on the result of these control experiments, we believe that the observed differences in TE abundances are a biological factor but not a technical [assembly] artefact.

As suggested, we have also added one more figure (Figure 4i) to show the normalized count of TE with respect to the chromosomal region analyzed. The result of this figure is described in L416-422 as *“Analysis of the chromosomal position of these elements revealed that different TE classes have dissimilar subtelomeric (e.g., within 300kb of chromosome ends) versus non-subtelomeric distributions with relatively more Gypsy, Afut1, Copia and DNA TcMar TEs located at non-subtelomeric regions, while* *LINE TEs are mostly found at subtelomeric regions* *(Figure 4h, Supplementary Table 4). When the size of the two genomic regions is taken into account, TEs are found at a relatively higher frequency at the subtelomeric regions (Figure 4i).”*

3) Some controls/data are missing: The manuscript does not report any biological replicates for the ChIP-seq experiments to assess the robustness of the observed patterns. It is essential to quantify the biological variation in these experiments (correlation between repeats), especially when quantitatively comparing levels of histone modifications such as shown in Fig. 1.

**Response:** The ChIP-seq and RNA-seq experiments in this manuscript were performed in two and three biological replicates, respectively. The replicate data is highly correlated (see Supplementary Figure 9) and all reported results could be observed in both replicates. The correlation values between sample replicates have been added to a new Supplementary Figure 9. The description of replicates and the new Supplementary Figure 9 is now given in the Methods section L789-791: *“All ChIP-seq experiments were performed in two biological replicates, and all reported patterns were observed in both replicates (Supplementary Figure 9a, b).”* and L851-853: *“All RNA-seq experiments were performed in three biological replicates, which are highly correlated (Supplementary Figure 9c).”*

4) The manuscript reports on potential centromeric regions using sequencing read coverage as evidence (L298). Are these locations identical to the ones reported by Fedorova et al. 2008? To firmly establish centromeric positions, ChIP-seq experiments targeted at CenH3/CENPA are essential.

**Response:** We are sorry that this information about centromeric regions was not given in the original manuscript. Yes, we had indeed used those centromeric locations reported by Fedorova et al. 2008 and McDonagh et al. 2008. As the exact centromeric positions do not affect the interpretation of the results or affect any conclusion of the current manuscript and the centromeric positions are well-annotated on the reference genome, we respectfully did not perform the suggested CenH3/CENPA ChIP-seq experiments. Nevertheless, we have revised the label of the centromeric positions as “Annotated centromeres” to indicate that they are probable centromeric regions and refer to those H3K9me3 peaks as being “near” (rather than “at”) centromeric and pericentromeric regions.

5) Some important references are missing: The manuscript mentions (L109) that post-translational modification in A. fumigatus and their role in virulence and pathogenicity are unknown. However, several articles have started to explore these links in the couple of years, which need to be discussed and addressed (such as Palmer et. al 2008; Li et al. 2019; Lin et al. 2020).

**Response:** We are very sorry for missing the references. They are now cited and discussed in L132-134: *“In A. fumigatus, chromatin modifications have been shown to affect growth, conidiation, stress responses, biofilm formation and fitness during infection (Palmer et. Al, 2008; Li et al., 2019; Lin et al. 2020).”*

Further comments:

L67: the suggested contradiction between environmental diversity and the capacity of A. fumigatus to infect patients is not clear. Could the authors please clarify this point?

**Response:** This sentence has been removed.

L282: ‘corresponding to the Af293…’; this part of the sentence is redundant to line 279 and should be removed.

**Response:** The sentence has been removed.

L285: The authors observe an interesting relationship between gene expression levels and histone modifications, as previously reported in other fungal system. They also report on the location of suppressed genes (group 3) at H3K9me3 regions at telomeres. Telomeric regions have previously been shown to be transcriptionally dynamic during infections (McDonagh et al. 2008), and it would be very interesting to assess if these H3K9me3 marked genes are expressed during infection. Typically, H3K9me3 has been considered to mark obligatory heterochromatin and facultative heterochromatin such as H3K27me3 is absent in Aspergilli. Thus, this observation would be an interesting observation to be added to the paper.

**Response:** We thank the reviewer for the suggestion. The mechanism of heterochromatin silencing is indeed an interesting question, especially given that *A. fumigatus* lacks the H3K27me3-mediated heterochromatin system.

As suggested, we checked the expression change of H3K9me3 associated genes (e.g., genes located within 5 kb of a H3K9me3 ChIP-seq peak) during infection reported in the McDonagh et al. 2008 study. As shown in the figure below, there is no significant enrichment of up- or down-regulated genes among the H3K9me3 associated genes, regardless of whether they are located at subtelomeric regions or not. Therefore, there does not appear to have any obvious relationship between H3K9me3 modification and the regulation during infection.

We feel that this result does not provide any specific insight into the H3K9me3 function and heterchromatin regulation, and therefore, we did not include this analysis into the manuscript. Nevertheless, we agree with this reviewer that understanding how heterochromatin silencing is established in A. fumigatus is very interesting if both well-established mechanisms (H3K27me3 and H3K9me3) are not involved. Therefore, we added the following sentence in L643-649 to highlight this interesting situation: *“However, we found here that most of A. fumigatus SM BGCs are not marked by H3K9me3 at the genome-wide level and that the modification does not strictly represent BGC silencing. In other words, our observations suggest a minor role of H3K9me3 on SM BGC regulation. This raises an interesting question as to how is silencing of SM BGC achieved in A. fumigatus, given that the other major heterochromatic mechanism mediated by Polycomb Repressive Complex and H3K27 methylation is also not present (Connolly et al., 2013).”*

L632: The manuscript discusses potential reasons for the presence of H3K4me3 is not strictly linked to increased gene expression (L631). One additional explanation is related to the authors’ analyses of only a single activating mark, while many more are chromatin marks associated with active genes are known in Aspergilli and other fungi (see Collemare et al. 2019). Thus, an additional explanation could indeed be, as suggested by the authors, that H3K4 is absent but replaced by another activating mark.

**Response:** We thank the reviewer for this alternative possibility. We have revised the discussion to include this in L618-630 “*Unexpectedly, a group of well-expressed genes (based on the presence of mRNAs) including many transporter genes were not marked by this modification. One explanation for this is that the transcription of those genes does not involve active histone modification as shown for mammalian developmental genes (Pérez-Lluch et al., 2015). Alternatively, those genes are controlled by a unique chromatin regulatory mechanism that involves other histone modifications but not H3K4me3 (Collemare and Seidl, 2019). Yet another non-mutually exclusive explanation is related to the fact that ChIP-seq experiments, through formaldehyde crosslinking, capture events that occur at the time of cell harvest. Therefore, those transporter genes may be transcribed earlier before the actual experiment (i.e., during spore germination or the initial growth stage) but their mRNAs are stable enough until the point of experiment for detection by RNA-seq, whereas H3K4me3 was removed soon after transcription ceased.*”

L702: ‘tospeculate’ ◊ ‘to speculate’

**Response:** ‘tospeculate’ has been corrected to ‘to speculate’.

L836: The Nanopore genome assembly was finalized using Ragout with a publicly available reference genome as reference strain. Fungal isolates can differ considerably in the chromosomal makeup even between closely related isolates and the authors

**Response:** The reviewer did not finish the sentence and unfortunately we cannot understand the question. Since we have multiple independent validation on the missing Chr VIII region and considering a potential concern this reviewer had on the Nanopore analysis, we have decided to completely remove the Nanopore result from the manuscript. The removal does not affect any conclusion drawn in the manuscript.

L858: how were TE families defined? Did the authors predict them de novo or were these based on previously generated consensus sequences?

**Response:** TEs were defined and identified using the program RepeatMasker-4.1.1 (Smith *et al*., 2015). As described in the program, the analysis is based on previously generated consensus sequences. To clarify this information, we have now added the following sentences in the result and method sections:

- L370-373 “*To systematically identify TEs, we employed the* *RepeatMasker program to search the Af293 and CEA10 reference genomes against an open database of TE profile HMM models and consensus sequences (Smith et al., 2015).*”;
- L383-384 “*RepeatMasker analysis of the Af293 and CEA10 reference genomes identified 539 and 654 TEs, respectively (Supplementary Table 4).*”;
- L395-396 “*To determine which class of TEs are modified by H3K9me3 in A. fumigatus, we used the RepeatMasker software to further sub-classify the identified TE elements.*”
- L858-859 *“TEs were identified using RepeatMasker-4.1.1 (http://www.repeatmasker.org), which reports Repeats and TEs according to previously established TE consensus.”*

L866: the sentence reads odd and needs to be rephrased

**Response:** The sentence has been changed to “Raw sequencing reads of histone ChIPseq experiments were checked for quality using FastQC (http://www.bioinformatics.babraham.ac.uk/projects/fastqc/) and aligned to *A. fumigatus* reference genomes (Af293 [genome version s03-m05-r06] or CEA10 [A1163]) using Bowtie2 (version: 2.2.9) using parameters that allow no mismatch (Langmead, Trapnell, Pop, & Salzberg, 2009).”

Fig. 1a: the ChIP-seq signal higher for one of the isolates compared with the other. To better compare between isolates the authors need to normalize the signal

**Response:** All ChIP-seq signal values (including those of H3 ChIP-seq) presented in the original submission are normalized to total sequenced reads. We have now added the normalization information to the Methods section in L788-789 as “*ChIP-seq signals were normalized to total sequence reads.*”.

The reviewer has rightly pointed out that the H3 ChIP-seq signal in TSS region is higher and narrower in CEA17 than Af293. We also noticed this observation, but we do not understand the phenomenon and did not comment on this point. We would like to keep the results here if this reviewer has no objection, in case the observation is relevant for some readers.

Fig. 2a: it would be clearer if the H3K9me3 occupancy patterns would be displayed in relationship to scaled chromosomes sizes (i.e., first 10%, 20%, 30%, etc.) to account for differences in chromosome size (chr1 vs. chr8).

**Response:** The intended purpose of Figure 2a is to show that H3K9me3 modification occurs at the chromosome ends, which are not influence by chromosome sizes. This is why we show the H3K9me3 occupancy only at the 100kb from chromosomal ends. The overall occupancy over entire chromosomes (i.e., taking into account of different chromosome sizes) is presented in Figure 2e (Figure 2g and h in the original submission). Hence, we prefer to keep these two figure panels in their current formats. Nevertheless, in consideration of this comment, we have revised Figure 2a to focus on subtelomeric (e.g., 300 kb from chromosome ends) and centromeric regions. Figure 2e has also been simplified showing the locations of H3K9me3 peaks and BGCs only, and arrows connecting corresponding chromosomes between Af293 and CEA17 have been added to help readers compare the results.

Reviewer #2

The manuscript submitted by Colabardini and Wang et al., provide a comparison of two isolates of Aspergillus fumigatus, termed Af293 and CEA17, the causal agent of Invasive Pulmonary Aspergillosis (IPA). To explore differences between the strains, the authors conducted ChIP-seq for H3K4me3 and H3K9me3. The authors mainly addressed how domains of H3K9me3 associated with secondary metabolite clusters in the genome and transposable elements. The authors report that TEs were significantly different between the two lines, with a major difference being Af293 having more LINE retrotransposons. The authors provide some comparative analysis that this increase results in more genomic instability for line Af293. The authors report on a derivative strain of Af293, termed Af293GG that contains a ~320kb deletion, altered SM profile, an amplified region and altered morphology.

While the paper is interesting, it suffers from imprecise characterization and breaks in the logical flow.

Major concerns:

1) Is there a better control whereby the authors can support their claim that using the Af293 genome for CEA17 data results in accurate conclusions? The authors only report a similar mapping rate, but this is a weak argument. The authors can bolster the methodological approach, and thereby the findings, by providing more evidence the results are not because of using a single reference.

**Response:** We thank this reviewer for this critical, reasonable comment, which is also raised by Reviewer #1. A few control analyses have been added to address the differences in genome assembly quality. Specifically, we compared the size of reference genomes of Af293, CEA10 and 79 other *A. fumigatus* isolates. Compared to the fully assembled Af293, the CEA10 reference genome is at least 99% complete. It is also among the most completed genomes among the analyzed genomes. Therefore, we believe that the reference genome, despite being partial, is of high quality with only some very small gaps. The comparison is described in L194-199 as “*Comparison of the size of CEA10 reference genome (A1163) with those of Af293 and other A. fumigatus isolates (n = 79 from Takahashi-Nakaguchi et al., 2015; Zhao et al., 2021) shows the CEA10 reference genome size is greater than most isolates (Supplementary Figure 1a), and comparison to the full assembly of Af293 genome suggests that the CEA10 reference probably covers more than 99% of A. fumigatus genome (Supplementary Table 1), and therefore, has sufficient coverage for analysis and comparison.*”

In addition, to rule out the possibility that the observation is due to a technical (assembly) factor, we have also performed Whole-Genome Re-sequencing by Illumina for Af293 and CEA17, mapped the data to their respective genomes and counted TE elements from the unmapped reads. The rationale behind this analysis is that if there were many TEs missing from the partial CEA10 reference genome, many unmapped reads from the mapping of Whole-Genome Re-sequencing data (which covers the entire genome) would be expected to carry TEs and the number would be much higher compared to the control experiment using Af293 Whole-Genome Re-sequencing data. The result of this analysis is described in L373-382 as “*Since the CEA10 reference genome is not fully assembled, we first confirmed that the existing sequence and assembly is suitable for TE analysis. To this end, Illumina sequencing for the Af293 and CEA17 isolates was performed, and the raw sequences were mapped to both reference genomes separately. If there were many TEs missing from the partial CEA10 assembly, a high number of unmapped reads (from mapping CEA17 data to the CEA10 genome reference) is expected to contain TE sequences. When using the complete Af293 reference genome and Af293 data as a control, the comparison showed that the numbers of TEs from mapping the Af293 and CEA17 Illumina sequences to their respective genomes are similar (Supplementary Figure 4a), indicating that the CEA10 reference genome is just as good as the Af293 one for TE analysis.*”

We believe that these additional control experiments along with those control analyses in the original submission can support our claims.

2) The analysis is not precise in places. Phrases from the authors such as ‘’ the distribution of H3K9me3 peaks [..] of the two isolates was somewhat different’. This is too colloquial and lacks from formal/rigorous analysis. The following conclusion ‘Therefore, these results reveal dissimilarities in the pattern of H3K9me3 deposition on Af293 and CEA17 genomes’ is difficult to assess because the analysis itself is not complete.

**Response:** We have carefully rewritten the manuscript paying attention to the style of writing and have removed the sentence “*the distribution of H3K9me3 peaks [..] of the two isolates was somewhat different*”. In addition, we have added new analysis and revised figures (e.g., Figure 2b, d, e; Supplementary Figure 2d; Supplementary Figure 3b, c) to better show the direct comparisons between the isolates. In particular, Figure 2e has been simplified to focus on the locations of H3K9me3 modifications and BGCs at the genome-wide level (e.g., covering all eight chromosomes) in Af293 and CEA17. The four corresponding chromosomes between the two isolates are indicated by arrows for easy comparisons. A new figure (Supplementary Figure 2d) has been added to show select genomic loci with different H3K9me3 modifications. Furthermore, figures in the original Supplementary Figure 3 have been re-arranged to show H3K9me3 modification at the common BGCs of Af293 and CEA17. Thanks to this comment, these revisions have significantly improved the presentation of our observations and the manuscript.

3) There does not appear to be substantial evidence that supports the conclusion that Af293 and CEA17 have different epigenetic patterns (section lines 260-324). For instance, it is not clear what is being shown in Figure2 a, and the analysis is not systematic or complete. Figure 2g and h are just pictures and it is difficult to draw a conclusion about how similar or different they are. The conclusion seems to be more concreate than the supporting data.

**Response:** We regret to know that Figure 2a was confusing. The main purpose of the original Figure 2a (which is a heatmap showing H3K9me3 ChIP-seq signal at the indicated regions) is to show the enrichment of H3K9me3 modification at the ends of Af293 chromosomes (100 kb boxes on both edges) and to contrast with 100 kb regions within the body of chromosomes. This figure is not intended for a systemic analysis. On the other hand, the original Figure 2g and h was meant for showing the result of a systematic analysis covering all chromosomes. Considering this reviewer’s comment, we realized that the original Figure 2g and h probably contains too much information. We have now revised the figure (now is Figure 2e) to display the chromosomal locations of H3K9me3 peaks and BGCs only. To better clarify the result shown in Figure 2a, it is now modified to show the H3K9me3 ChIP-seq signals over the 300 kb regions of chromosome ends (which include both telomeric and sub-telomeric regions) and the centromeric regions. Hence, the figure is now consistent with what is being described in the text. Moreover, a new figure (Supplementary Figure 2d) has been added to show select common genomic loci (i.e., exists in both isolates not affected by sequence variation) with different H3K9me3 modification between Af293 and CEA17. The figures in the original Supplementary Figure 3 have also been re-arranged and re-labeled for better comparisons between the two isolates. We believe that the revised figures should allow systematic and complete comparisons between the two isolates and help to improve on the manuscript.

In addition, we have also toned down on our conclusions; specifically, we removed phrases such as “Af293 and CEA17 have different epigenetic patterns” and “significant differences” and simply describe the various observed differences.

4) A similar problem for the SM analysis, it is not complete, and the data shown do not support the conclusions. How does figure 3a show that two clusters have a similar H3K9me3 profile? Where is data showing the other SMs clusters do not have a similar profile? Are the SM clusters in strain CEA17 well assembled and identifiable?

**Response:** We are sorry that our text and figures failed to deliver the intended messages. Figure 3a shows one type of H3K9me3 modification patterns, namely modification within a given BGC cluster. It is in this regard that we refer the two clusters have a similar profile. The original Figure 3b and 3c are supposed to show that the other clusters do not have a similar “within cluster” pattern. The original Figure 3b shows another type of modification pattern – modification found outside of and adjacent to annotated BGCs border (i.e., not within cluster). The original Figure 3c shows that, in both isolates, many clusters are far from H3K9me3 and the distances between BGCs to the closest H3K9me3 peak are highly diverse, and therefore most BGCs do not have a “within cluster” or “adjacent” pattern. In addition, the figure also shows that the distances are not the same in the two isolates. To better illustrate and distinguish the two slightly different modification patterns, the subtitles “H3K9me3 modification within BGC” and “H3K9me3 modification outside BGC” have been added to Figure 3a and 3b, respectively.

As for whether the SM clusters in strain CEA17 were well-assembled and assigned, we did factor this into the analysis and searched for all BGC genes from both CEA17 and Af293 annotations in the two genomes. The results related to this analysis are described in L320-328 “*As shown previously (Fedorova et al., 2008), the genomic locations of most SM BGCs on the four largest scaffolds of CEA17 (n = 17) were similar to the corresponding chromosomes I, II, III, V of Af293 (n = 19) (**Supplementary Table 3), with a few exceptions (e.g., BGCs 9-16 whose genomic arrangement was inverted between CEA17 Scf_3 and Af293 chromosome III (Supplementary Figure 3a) and BGCs 27 and 33 that were located at different chromosomes in the two isolates) (Supplementary Table 3). To better analyse the situation, we mapped all identified H3K9me3 peaks to SM BGCs in the two isolates. There are 31 common SM BGCs in both Af293 and CEA17, and Af293 genome contains two additional SM BGCs (BGC 1 and 4) that are not present in CEA17 (Supplementary Table 3) (Lind et al., 2017).*”. Therefore, we believe that the analysis is sound and the observations made are reliable and that the revision can now better present the observations to support the conclusions.

5) Why does line 343 say ‘we found only two H3K9me3 peaks located within SM BGCs for both’, while line 359 says ‘Among the H3K9me3-associated clusters, five clusters (BGCs 8, 10, 16, 19 and 25) (Figure 3c) were commonly modified by H3K9me3 between the two isolates.’ ? Is this a difference between H3K9me3 being within the cluster versus close by? It is not clear.

**Response:** We are sorry for the unclear message. We had an unexpected observation that most BGCs do not have H3K9me3 modification, and we observed a few different modification patterns among the BGCs. Since we did not know whether there is any difference between the modification patterns from the mechanistic perspective, we try to be as descriptive as we could by classifying the H3K9me3 modification profile into a few groups based on the location of H3K9me3 ChIP-seq peaks with respect to annotated BGCs borders. This is why we separately mentioned the two and five BGCs that have H3K9me3 peaks found “within” or “outside of and adjacent” to the cluster borders, respectively. We have revised the text to clarify the observation in L340-348: “*We, therefore, extended the analysis to identify the nearest H3K9me3 peak for each SM BGC, and only 5 SM BGCs had an H3K9me3 peak located outside of and adjacent to the annotated cluster border in both Af293 (BGCs 4, 8, 19, 25 and 33) and CEA17 (BGCs 8, 10, 12, 19 and 25) (Figure 3b, c). Hence, together with the two BGCs with H3K9me3 peaks within the clusters (Figure 3a, Supplementary Figure 3b), only seven SM BGCs in each of the isolates (out of 33 and 31 SM BGCs in Af293 and CEA17, respectively) are modified by or located near H3K9me3 modified regions. Among these H3K9me3-associated clusters, five clusters (BGCs 8, 10, 16, 19 and 25) (Figure 3c) were common between the two isolates.*”. To better illustrate the two slightly different modification patterns, the subtitles “H3K9me3 modification within BGC” and “H3K9me3 modification outside BGC” have been added to Figure 3a and 3b, respectively.

Based on our gene expression analysis of BGCs genes, both patterns (and H3K9me3 in general) do not appear to contribute much, if any, to their regulation – at least based on the conditions we have analyzed here.

6) It is very difficult to put much faith in a TE comparison between a well assembled and a less well assembled genome. How does the better reference of Af293 affect the results? The detection of 5x more LINE elements in Af293 may be true, and some additional analysis is provided, such as a qPCR for a specific DNA segment. But then the authors make a significant logical leap with the concluding of the paragraph (lines 455-456), ‘these results imply that Af293 genome may have higher LINE-transposon activity and, hence, more unstable.’

**Response:** We thank the reviewer for this legitimate comment about the difference in the reference genomes used. Reviewer 1 also raises a similar concern. To address this, we have added a few control analyses. First, we compared the size of reference genomes of Af293, CEA10 and 79 other *A. fumigatus* isolates. Compared to the fully assembled Af293, the CEA10 reference genome is estimated to be at least 99% complete. It is also among the most completed genomes among the analyzed genomes. Therefore, we believe that the reference genome, despite being partial, is of high quality with only some very small gaps. The comparison is described in L194-199 as “*Comparison of the size of CEA10 reference genome (A1163) with those of Af293 and other A. fumigatus isolates (n = 79 from Takahashi-Nakaguchi et al., 2015; Zhao et al., 2021) shows the CEA10 reference genome size is greater than most isolates (Supplementary Figure 1a), and comparison to the full assembly of Af293 genome suggests that the CEA10 reference probably covers more than 99% of A. fumigatus genome (Supplementary Table 1), and therefore, has sufficient coverage for analysis and comparison.*”

In addition, to rule out the possibility that the observation is due to a technical (assembly) factor, we have also performed Whole-Genome Re-sequencing by Illumina for Af293 and CEA17, mapped the data to their respective genomes and counted TE elements from the unmapped reads. The rationale behind this analysis is that if there were many TEs missing from the partial CEA10 reference genome, many unmapped reads from the mapping of Whole-Genome Re-sequencing data (which covers the entire genome) would be expected to carry TEs and the number would be much higher compared to the control experiment using Af293 Whole-Genome Re-sequencing data. The result of this analysis is described in L373-382 as “*Since the CEA10 reference genome is not fully assembled, we first confirmed that the existing sequence and assembly is suitable for TE analysis. To this end, Illumina sequencing for the Af293 and CEA17 isolates was performed, and the raw sequences were mapped to both reference genomes separately. If there were many TEs missing from the partial CEA10 assembly, a high number of unmapped reads (from mapping CEA17 data to the CEA10 genome reference) is expected to contain TE sequences. When using the complete Af293 reference genome and Af293 data as a control, the comparison showed that the numbers of TEs from mapping the Af293 and CEA17 Illumina sequences to their respective genomes are similar (Supplementary Figure 4a), indicating that the CEA10 reference genome is just as good as the Af293 one for TE analysis.*”

We believe that these new results and the qPCR confirmation presented in the original manuscript provide strong supports to the result of TE comparison. Considering this and the other comments about toning down our conclusions, we have removed this sentence “*these results imply that Af293 genome may have higher LINE-transposon activity and, hence, more unstable*” from the manuscript.

7) The section using public RNA-seq data for the Af293 strain to check for chromosomal aberrations, detected as loss of read-mapping for contiguous regions, is quite interesting. The important control, similar analysis for strain CEA17, is present. What is not clear is how similar or different are the experiments that make up the two sets of data. Can the authors make a table that summarizes somehow the types of experiments that are captures by the two sets of data. That is, was a given RNAseq bioproject looking at growth and development, mutant analysis, in vitro growth, stress conditions, etc. For instance, data from PRJNA421149 appear to have significantly more lost regions than other data sets. Is the because of the strain used in that lab or because of the experimental conditions from that set of experiments? The alternative hypothesis that is not clearly tested is that the type of experiments that make up the two strains datasets are themselves very different and therefore influence the amount of genomic instability observed.

**Response:** We thank this reviewer for requesting this important information, which is now given in Supplementary data 3. We also looked into the nine datasets in PRJNA421149, which contains RNA-seq data of *A. fumigatus* obtained from mice, and found that these datasets indeed have the lowest number of mapped reads (<1M) (presumably due to relatively low amounts of fungal material in infected tissues). All these datasets have been removed from the analysis and Figure 6a, f and Supplmentary Figure 5 have been updated accordingly.

Regarding the alternative hypothesis, we have summarized the experimental conditions into a table in Supplementary Data 3 showing that the datasets used for Af293 and CEA17 analysis are not very different, ruling out the possibility that the observed result is due to distinct make up of the two strains datasets. This is described in L468-470 as “*Both isolates have largely similar datasets covering a range of experimental conditions such as defined media, mice infection, interactions with mammalian and bacterial cells (Supplementary Data 3).*”.

8) I do not think there is a very strong link between the data and the overall conclusion. (line 759-761) ‘Overall, our work emphasizes the importance of epigenetic modifications in A. fumigatus strain heterogeneity as evolutionary drivers of altered gene expression and genome stability.’ There is not a strong connection between the epigenetic data collected and the reported structural variation observed in the GG strain’s genome. There is not a rigorous description of epigenetic differences and how this relates to the SV differences. The authors seem to go from the ChIP data, to there are different TEs, to there are different genome stability between the lines. But there is no data for causation. This is needed to ‘emphasize the importance of epigenetic modifications …. as evolutionary drivers’

**Response:** The reviewer is right that our results are suggestive. Since the main goal of this manuscript is to report the epigenetic and genetic heterogeneity among *A. fumigatus* isolates, we have not followed up on the experiments to demonstrate the causation between the observed events. In fact, this will be the subject of a follow up work. Nevertheless, to address this comment, we have now significantly rewritten the manuscript and have toned down our main conclusions. Below are a few major changes (which are the same as the response to Reviewer #1 comment 1):

1) The title is changed from “Chromatin profiling reveals genome stability heterogeneity in clinical isolates of the human pathogen Aspergillus fumigatus” to “Chromatin profiling reveals heterogeneity in clinical isolates of the human pathogen *Aspergillus fumigatus*” to emphasize on the findings of epigenetic and genetic heterogeneities between isolates.

2) The conclusion “Overall, our work emphasizes the importance of epigenetic modifications in *A. fumigatus* strain heterogeneity as evolutionary drivers of altered gene expression and genome stability” is deleted from the manuscript.

3) The manuscript has been significantly rewritten to tone down the conclusions according to this reviewer’s comment including (but not limited to) the following examples to just name a few changes:

- Removed sections like “Suggesting that some of them may not be subjected to H3K9me3 silencing since the LINE-transposons are the most active RNA transposons and have a major impact on the genome stability (Bourque *et al*., 2018), These results imply that Af293 genome may have higher LINE-transposon activity and, hence, more unstable”;
- Renamed the title of the sub-section from “Evidence for Af293 having a higher level of genome instability” to “Evidence for Af293 having an increased number of chromosomal rearrangement events”;
- Replaced the sentence “…the induced LINE-transposon activity may be responsible for the chromosomal loss” with “…the induced LINE-transposon activity may be linked to the chromosomal loss”;
- Replaced the sentence “…evidence that the Af293 genome is relatively unstable compared to CEA17” with “…evidence that the Af293 chromosomes are more prone to rearrangements compared to CEA17”;
- Added the following two paragraphs to the Discussion section to clarify the suggestive nature of our findings and highlight the need for additional experiments to confirm the causative links: “*Another interesting finding is that Af293 contains significantly more LINE-transposons than the CEA17 isolate, which was also reported in a previous study (Huber and Bignell, 2014). Our analysis revealed that a high percentage (~70%) of LINE-transposons in Af293 were not subjected to H3K9me3 modification (Figure 5e) and that the LINE transposon sequences in Af293 contain relatively much less mutations than in those in CEA17 (Figure 5b, c). Based on these, it is tempting to speculate higher transposon activities in the Af293 isolate. In support of the speculation and consistent with the association of transposons activity with chromosome rearrangement and genome instability in different organisms (Zeller et al., 2016; Miousse et al. 2015; Moller et al., 2019; Kondo et al., 2008; Peters et al., 2001; Peng et al, 2009), we indeed detected gross chromosome alteration events including a chromosomal loss hotspot in Chr VIII in Af293 strains of different laboratories but not from different CEA17 strains. This circumstantial evidence leads us to a proposal that the higher number of LINE transposons in Af293 may be linked to the increased incidents of chromosomal rearrangements observed among Af293 strains. It will be interesting to experimentally confirm this (e.g., by microevolution experiments in the absence of H3K9me3 modification and/or with increased transposon activity) and to compare transposon numbers and activities between different clinical isolates, considering chromosomal rearrangement has been shown as a virulence mechanism for the adaptation of pathogenic Candida species to different host niche environments (Poláková et al. 2009, Selmecki et al., 2006).*”and “*The identification of the Af293GG and other Af293 variants used by different laboratories implies additional levels of complexities in the heterogeneities of A. fumigatus, emphasizing the fascinating ability of this important fungal pathogen to evolve new growth and fitness characteristics that could influence virulence. Our study suggests a potential link between epigenetic heterogeneity (e.g. H3K9me3 modification), TEs, genome instability and genetic heterogeneity. Confirming a causative relationship between these events and identifying additional mechanisms that drive the heterogeneities will guarantee insights into A. fumigatus virulence.*”

Minor:
The patterns discussed in panel 1c are clear, but the use of the term ‘positively correlate’ (line 213) has a specific statistical meaning, and no correlation is shown. Either a correlation should be computed and reported, or the term changed. This occurs again at other parts of the manuscript.

**Response:** Statistics have been computed and added to Fig. 1d and other parts of the manuscript.

There are some duplicated sentences and other English mistakes in the MS. It needs another round of proof-reading.

**Response:** We have carefully edited and proofread the manuscript.

Reviewer #3

In this manuscript, Colabardini, Goldman and colleagues focus on molecular genetics of the two standard A. fumigatus strains, Af293 and CEA17 (derivative of CEA10). First, the authors perform ChIP-seq experiments to characterize genome-wide distributions of H3K4me3 and H3K9me3. Here the authors find that not all actively transcribed genes are associated with H3K4me3, while not all silent secondary-metabolite clusters are associated with H3K9me3. These observations will surely pave the way for more detailed studies in the future. Second, the authors also analyze TE content and discover that TEs are more prevalent in Af293, where they may actually drive substantial genome instability. To identify instances of large deletions the authors make a clever use of publicly available transcriptomic data-sets.

**Response:** We thank the reviewer for the positive comment.

Overall, this manuscript presents a collection of interesting findings and follow-up experiments concerning the molecular genetics of A. fumigatus.

While the authors identify a clear difference in TE content between the two strains, it would be interesting to see further analysis that might explain this difference. For example, RIP was still reported to occur in A. fumigatus (doi 10.1134/S0026893307060039), despite its largely asexual lifestyle. Is it possible that a lager proportion of TEs in CEA17 (as compared to Af293) are inactivated by RIP?

**Response:** We thank the reviewer for this great suggestion that our data can offer additional independent evidence for RIP (repeat-induced point mutation) and provide insights into whether RIP may be involved in genome defense against TEs in *A. fumigatus*. We have analyzed the type of mutations reported in the original submission and found that the mutations in CEA17 LINE TEs (which has accumulated many more mutations than those in Af293) are largely C:G to T:A mutations (94.9%), which are the exact type of mutation introduced by the RIP process. On the other hand, C:G to T:A mutations are only found in 6.8% of the mutations on the LINE TEs in Af293. This observation not only indicates an active RIP process for genome defense against TEs in *A. fumigatus*, but also suggests heterogeneity in this process between CEA17 and Af293. The result is described in L439-447 of the Result section as the following: “*Notably, we observed a remarkable difference in the number of mutations among the LINE TEs of Af293 and CEA17 as revealed by comparisons to the consensus DNA sequence LINE TE. The LINE-transposons in the Af293 genome accumulated less mutation as compared to CEA17 (Figure 5b, c), indicating potentially functional transposon activity from some LINE transposons in Af293 if expressed. The type of mutations also differ between the two isolates with the majority of the mutations (~95%) in the LINE transposons in CEA17 being C:G to T:A (Figure 5d and Supplementary Table 5), which indicates transposon inactivation by the repeat-induced point mutation (RIP) process (Selker 2002), while this was not the case for Af293.*”

The following paragraph is also added to the Discussion section in L680-694: “*Fungi utilize a number of genome defense mechanisms for protection against TEs including RIP that was first identified in N. crassa (Aramayo and Selker, 2013). Although RIP has not been demonstrated in A. fumigatus, an early study had implicated its existence and protection against TEs (Neuveglise et al., 1996). It is noteworthy that the mutations observed at the LINE transposons in CEA17 (e.g., >95% mutations are C:G to T:A transitions) is highly indicative of transposon inactivation by RIP (Selker, 2002). Remarkably, the Af293 isolate has a strikingly contrasting mutational profile in which only around 7% of the mutations are C:G to T:A transitions. Consistent with RIP functioning in conjunction with H3K9me3 heterochromatic silencing (Gladyshev, 2021), H3K9me3 modification was also found on top of all CEA17 LINE transposons but not on Af293 LINE transposons. If RIP indeed operates in A. fumigatus, the interesting heterogeneities in their LINE mutational profiles potentially suggests different levels of genome defence by RIP between the two isolates. In N. crassa, the RIP process occurs during the sexual stage, and if it is also conserved in A. fumigatus, the result may suggest another mutually non-exclusive possibility that the two isolates have different levels of sexual activities.*”

Minor issues:

Line 563: extra ‘.’ after ‘above’

**Response:** This has been corrected.

Line 685: space missing in ‘heresuggest’

**Response:** This has been corrected.

Line 702: space missing in ‘tospeculate’

**Response:** This has been corrected.

**Have all data underlying the figures and results presented in the manuscript been provided?**

Large-scale datasets should be made available via a public repository as described in the *PLOS Genetics* [data availability policy](http://journals.plos.org/plosgenetics/s/data-availability), and numerical data that underlies graphs or summary statistics should be provided in spreadsheet form as supporting information.

Reviewer #1: Yes

Reviewer #2: **No:** From what I could tell, there is not an SRA entry for PRJNA728823, so I cannot tell if the data are available.

Reviewer #3: Yes

**Response:** All data have been deposited the NCBI SRA database and will be made publicly available upon acceptance.
