# Supplementary material for: Chromatin profiling reveals heterogeneity in clinical isolates of the human pathogen Aspergillus fumigatus
Source: PLoS Genet. 2022 Jan 10;18(1):e1010001. doi: 10.1371/journal.pgen.1010001 (PMC8782537; doi:10.1371/journal.pgen.1010001)
Supplement: Attachment — Submitted filename: Answer to the Reviewer.docx [file pgen.1010001.s028.docx]

Answers to the Reviewer #1

1) The revised manuscript 'Chromatin profiling reveals heterogeneity in clinical isolates of the human pathogen Aspergillus fumigates' successfully addresses most of my raised comments. As previously mentioned, the manuscript reports on an exciting suite of novel data that will be interesting for researchers working on Aspergillus, chromatin biology, and genome evolution.

Answer: We thank the reviewer for all the suggestions and comments that dramatically improved the manuscript. We are very thankful to the reviewer.

I still have few minor remarks to be considered by the authors prior to publication:

2) The abstract does not explicitly link the differences in chromatin profiles to differences in TE number/activity and ultimately genetic heterogeneity. This link is clearer laid out in the 'Authors summary' (L78) and I would encourage the authors to adjust the Abstract accordingly

Answer: We have changed the abstract by rewriting a sentence to address this heterogeneity linkage with diferences in TE number/activity (Lines 60 to 61): “..and the differences are correlated with H3K9me3 modifications and higher genomic variations among strains of Af293 background.”

3) L192: Could the authors briefly explain/mention the differences between CEA10 and CEA17?

Answer: CEA10 is a pyrimidine auxotrophic derivative of CEA17, thus we changed the sentence in line 157 to “Af293 and CEA17 (a pyrimidine auxotrophic derivative isolate of the clinical isolate CEA10)” and added a new reference [59] (Bertuzzi et al., 2021) to clarify this point.

4) Fig. 3 shows that some H3K9me3 peaks occur outside and inside of BGC. Could the authors please add the TE track to these pictures to show that these peaks are (not) associated with transposons and other repeats that could explain the different patterns, i.e., association to TEs rather than association to BGCs

Answer: The TE tracks at these BGCs loci were added in Fig 3A, B and S3B Fig accordingly, and we modified the text *“*It is noteworthy that the H3K9me3 modifications found at some (but not all) BGCs also associated with the presence of TEs (Fig 3A, B), and different TE distribution may also explain the different H3K9me3 modification patterns at some BGCs between the two isolates (e.g. BGC21; S3B Fig)." in lines 386 to 389.

5) The manuscript reports that isolated TEs do not correlate with H3K9me3. However, only peaks called by MACS2 were used rather than assessing the quantitive H3K9me3 levels above the background. This could still reveal an association of this chromatin mark to TEs outside of TE cluster. Thus, I would encourage to briefly address and test this possibility.

Answer: We showed in the original Fig 4B that isolated TEs tend to lack H3K9me3 modification compared to TE clusters from genome browser inspection. To test the association of TE with H3K9me3 modification systematically and as suggested by this reviewer, we quantitated the H3K9me3 level at each TE region. Hence, a new figure Fig 4C was added and the description “Indeed, some of the H3K9me3 peaks overlap with several TEs (Fig 4A, B) and strong H3K9me3 ChIP-seq signals were often found at or near TEs located in clusters (Fig 4B, indicated by arrow), while most isolated TEs have background levels of H3K9me3 (i.e., no or non-detectable modification) (Fig 4C). Systematic analysis showed that about 40% of H3K9me3 peaks called from MACS2 were found at or near TE(s) (Fig 4D, left panel) and around half (45%) of TEs were associated with H3K9me3 modification (Fig 4D, right panel), when considering a window of 4 kb spanning the given feature.” was added to the text in lines 379 to 386.

6) L691: the authors discuss the occurrence of RIP and its link to the sexual cycle. Recent work in Z. tritici (Moeller et al. 2021) discusses a process that similar to RIP leads to C->T mutations but operates during mitosis, which might be relevant to mention and discuss here as well.

Answer: This is an excellent suggestion. We have added a discussion about this to the manuscript (lines 682 to 685): “Alternatively, the C:G to T:A transitions may be due to a DNA methyltransferase-mediated mechanism that occurs during mitosis as found for the plant fungal pathogen Z. tritici [101]. Regardless of the mechanism, our result indicates differential TE inactivation activities between A. fumigatus clinical isolates.”

7) L703: FtmD should be italic

Answer: This was now changed (line 833)
